# Characterization of *Carotenoid Cleavage Oxygenase* Genes in *Cerasus humilis* and Functional Analysis of *ChCCD1*

**DOI:** 10.3390/plants12112114

**Published:** 2023-05-26

**Authors:** Chunzhen Cheng, Rui Yang, Lu Yin, Jianying Zhang, Limin Gao, Rong Lu, Yan Yang, Pengfei Wang, Xiaopeng Mu, Shuai Zhang, Bin Zhang, Jiancheng Zhang

**Affiliations:** 1 College of Horticulture, Shanxi Agricultural University, Jinzhong 030801, China; ld0532cheng@sxau.edu.cn (C.C.); yangrui13934147962@163.com (R.Y.); yl18434764709@163.com (L.Y.); 18734448590@163.com (J.Z.); 18404968975@163.com (Y.Y.); 15110671026@163.com (X.M.); wwzs_1990@aliyun.com (S.Z.); jztgzhangbin@163.com (B.Z.); 2 Agricultural Technology Extension Service Center of Qianyang County, Baoji 721199, China; 13466896541@163.com; 3 Rural Revitalization Bureau of Pu County, Linfen 041200, China; llrr0624@163.com

**Keywords:** *Cerasus humilis*, carotenoid cleavage oxygenase, carotenoids, apocarotenoids, functional analysis

## Abstract

Carotenoid cleavage oxygenases (CCOs) are key enzymes that function in degrading carotenoids into a variety of apocarotenoids and some other compounds. In this study, we performed genome-wide identification and characterization analysis of *CCO* genes in *Cerasus humilis*. Totally, nine *CCO* genes could be classified into six subfamilies, including carotenoid cleavage dioxygenase 1 (CCD1), CCD4, CCD7, CCD8, CCD-like and nine-*cis*-epoxycarotenoid dioxygenase (NCED), were identified. Results of gene expression analysis showed that *ChCCOs* exhibited diverse expression patterns in different organs and in fruits at different ripening stages. To investigate the roles of *ChCCOs* in carotenoids degradation, enzyme assays of the ChCCD1 and ChCCD4 were performed in *Escerichia coli* BL21(DE3) that can accumulate lycopene, β-carotene and zeaxanthin. The prokaryotic expressed ChCCD1 resulted in obvious degradation of lycopene, β-carotene and zeaxanthin, but ChCCD4 did not show similar functions. To further determine the cleaved volatile apocarotenoids of these two proteins, headspace gas chromatography/mass spectrometer analysis was performed. Results showed that ChCCD1 could cleave lycopene at 5, 6 and 5′, 6′ positions to produce 6-methy-5-hepten-2-one and could catalyze β-carotene at 9, 10 and 9′, 10′ positions to generate β-ionone. Our study will be helpful for clarifying the roles of *CCO* genes especially *ChCCD1* in regulating carotenoid degradation and apocarotenoid production in *C*. *humilis*.

## 1. Introduction

Carotenoids, a subgroup of isoprenoids that typically contain 40 carbons and abundant conjugated double bounds, are the most conspicuous pigments and the most widely distributed secondary metabolites in plants [1,2]. They play vital roles in photosystem assembly, light harvesting and photoprotection and contribute greatly to the pigmentation, scents and flavors formation and stress responses of plants [1]. Carotenoids are important precursors of a large number of apocarotenoids and some other compounds, such as geranial, α-ionone, β-carotene, geranylacetone, farnesylacetone, pseudoionone, abscisic acids (ABA) and strigolactones (SL) [3]. It is noteworthy that these carotenoid-derived apocarotenoids have been proven to contribute greatly to diverse plant biological processes by acting as pigments, volatiles, signals and phytohormones, and so on [2].

Carotenoids can be cleaved into apocarotenoids by carotenoid cleavage oxygenases (CCOs), lipoxygenases, peroxidases and reactive oxygen species [2]. Among them, CCO-mediated carotenoid degradation is the main focus of attention for carotenoid degradation research. Depending on whether their substrates are epoxidized, CCOs can be divided into nine-cis-epoxycarotenoid dioxygenases (NCEDs) and carotenoid cleavage dioxygenases (CCDs) [4]. Ever since the first discovery of a maize *CCO* gene [5], a large number of plant *CCOs* have been identified and functionally studied. In the model plant Arabidopsis, the nine *AtCCO* members are divided into five subfamilies, including CCD1, CCD4, CCD7, CCD8 and NCED [4]. The classifications of CCOs in other plant species are mostly referred to as Arabidopsis or are based on sequence similarities, cleavage property, cleavage sites and substrate accessibility [3,6]. In addition to the five main CCO subfamilies, CCD-like, CCD2 and zaxinone synthase (ZAS) subfamily members were also identified in certain plant species [7,8,9].

Pieces of evidence have revealed that the functions of CCOs from different subfamilies varied a lot. For example, the CCD1, CCD2 and CCD4 subfamily members are reported to be involved in the biosynthesis of aroma-, flavor- and color formation-related apocarotenoids [10]; the CCD7 and CCD8 subfamily members are closely related to the SL biosynthesis [11]; the NCED members play a certain role in the growth and development of plants and are involved in ABA biosynthesis and [4] members from other subfamilies were found to be of diverse roles in the biosynthesis of some other carotenoids-derived compounds [6,12,13]. The functions of CCD1 and CCD4 subfamily members in carotenoid degradation and apocarotenoid production were the most widely studied in higher plants [14,15]. Research has revealed that CCD1 enzymes lack plastid localization peptides and are generally cytoplasmic [6]. Their cleaved products are usually responsible for the volatile formation [16,17], and their roles in controlling β-ionone generation have been reported in many plants [18,19,20,21]. Meanwhile, CCD4 enzymes are plastid-located, and their substrate specificity is generally higher than CCD1s [22]. Citrus CCD4 can cleave β-carotene, β,β-cryptoxanthin and zeaxanthin into apocarotenoids [23]. Grape VvCCD4 can cleave δ-carotene and lycopene to produce α-violonone and geranylacetone, respectively [24]. And Chrysanthemum CmCCD4a can cleave β-carotene into β-ionone, which is important for the flower pigmentation [25].

The Chinese dwarf cherry (*Cerasus humilis*) is a perennial woody fruit tree native to China [26]. Comparative genomic studies revealed that *C. humilis* shared a very close relationship with some fruit trees from the *Prunus* genus, such as *P*. *persia* and *P*. *armeniaca* [26]. The fruits of *C*. *humilis* contain a variety of carotenoids and their derived compounds [27]. Up until now, however, the *CCO* gene family in *C. humilis* has not been systematically studied. In this study, whole genome-wide identification and characterization of *C. humilis CCO* genes was performed. The expression patterns of *ChCCO* genes in different tissues and organs and in fruits at different ripening stages were studied using transcriptome data and quantitative real-time PCR analysis (qRT-PCR). Moreover, to uncover the functions of *ChCCO* genes in carotenoids degradation, *ChCCD1* and *ChCCD4* genes were cloned, inserted into prokaryotic expression vector pET-28a and subjected to enzyme assays in *Escerichia coli* BL21(DE3) that can accumulate lycopene, β-carotene and zeaxanthin. The results obtained in this study will provide a basis for understanding the roles of *CCO* genes in regulating carotenoid degradation and apocarotenoid production in *C. humilis*.

## 2. Results

### 2.1. Identification and Characterization of ChCCOs

**Figure 1:**
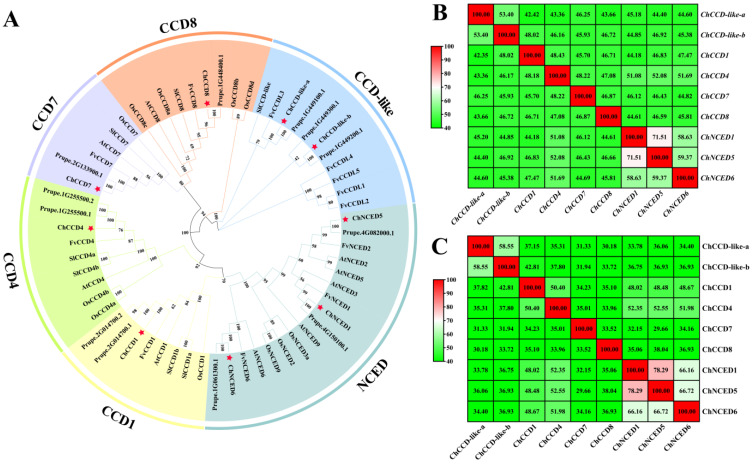
Phylogenetic analysis results (**A**) of CCO proteins from *Cerasus humilis* (Ch), *P. persica* (Pp), *Fragaria vesca* (Fv), *Solanum Lycopersicum* (Sl), *Oryza sativa* (Os) and *Arabidopsis thaliana* (At), and nucleotides (**B**) and proteins (**C**) similarity analysis results of ChCCOs. CCD1, CCD4, CCD-like, CCD8, CCD7 and NCED are six subfamilies of CCOs. Red stars in A represent *C. humilis* CCD members. In B and C: the redder the color, the higher the similarity; the greener the color, the lower the similarity.

Totally, we identified nine CCOs from the *C. humilis*. Phylogenetic analysis revealed that they can be classified into six subfamilies, including CCD-like, CCD1, CCD4, CCD7, CCD8 and NCED (Figure 1A). Among these subfamilies, the NCED subfamily consisted of three members (ChNCED1, ChNCED5 and ChNCED6), the CCD-like subfamily contained two members and the other subfamilies each contained only one member. Sequence similarity analysis revealed high similarities among ChNCEDs (Figure 1B). The similarity between *ChNCED1* and *ChNCED5* was about 71.51%, and their similarities with *ChNCED6* were about 58.63% and 59.37%, respectively. Similarities among their encoded proteins ranged from 66.16% to 78.29% (Figure 1C). Moreover, *ChCCD-like-a* and *ChCCD-like-b* shared a similarity of about 53.4%, and their encoded proteins shared a similarity of about 58.55%. Plant CCOs usually contained four conserved histidine active sites and three semi-conserved second shell glutamate residues [28]. Consistently, all the ChCCOs contained four conserved histidine and three conserved glutamate residues (Figure S1).

The coding sequence (CDS) length of *ChCCOs* ranged from 547 bp (*ChCCD1*) to 702 bp (*ChCCD-like-a*). Their encoded proteins consisted of 547~702 amino acids with their molecular weight ranging from 61777.14 Da (ChCCD1) to 78364.26 Da (ChCCD-like-a) and their theoretical isoelectric point (pI) ranging from 5.44 (ChCCD-like-a) to 6.97 (ChNCED6) (Table 1). All the ChCCOs were predicted to be hydrophilic proteins (GRAVY < 0), and, except for ChCCD7, ChNCED1 and ChNCED5 (with instability index > 40), all other ChCCOs were stable proteins (Table 1). Subcellular localization analysis revealed that ChCCOs were mainly located in the cytoplasm and chloroplast.

### 2.2. Chromosome Location and Synteny Analysis of ChCCOs

Chromosome location analysis revealed that *ChCCOs* were located in three chromosomes of *C. humilis* (Table 1 and Figure S2), including five members (*ChCCD-like-a*, *ChCCD-like-b*, *ChCCD4*, *ChCCD8* and *ChNCED6*) in Chr1, two members (*ChNCED1* and *ChNCED5*) in Chr3 and two members (*ChCCD1* and *ChCCD7*) in Chr5. Synteny analysis revealed that *ChCCD-like-a* and *ChCCD-like-b* were tandem duplicated genes (Figure S2).

### 2.3. Conserved Motifs in ChCCOs and Gene Structures of Their Corresponding Genes

Totally, we identified ten conserved motifs from the nine ChCCOs (Figure S3A). Among them, ChCCD-like-b, ChCCD1, ChCCD4, ChNCED1, ChNCED5 and ChNCED6 contained all the 10 conserved motifs. ChCCD-like-a did not contain Motif 1 and Motif 8, but it had two of Motif 10. ChCCD7 contained six motifs, including two of Motif 5 and one each of Motif 4, Motif 6, Motif 7 and Motif 8. And ChCCD8 only contained one each of Motif 2, Motif 3, Motif 4, Motif 6 and Motif 8.

Gene structure analysis results showed that, except *ChNCED1* and *ChNCED6*, all *ChCCOs* had introns (Figure S3B). The number of introns in *ChCCD-like-a* was the largest (19), followed by *ChCCD1* (14) and *ChCCD-like-b* (11). *ChCCD8* and *ChCCD7* had seven and five introns, respectively. And *ChCCD4* and *ChNCED5* both contained two introns.

### 2.4. Promoter Analysis of ChCCOs

The cis-acting elements in the promoter regions of *ChCCOs* were analyzed (Figure S4). Results showed that, in addition to the abundant light responsive, core promoter elements TATA-box and CAAT-box and growth and development-related elements, the *ChCCOs*’ promoters also contained a variety of phytohormone- and stress-responsive elements.

Ten types of phytohormone-responsive elements involving six phytohormones (ABA, MeJA, auxin, gibberellin (GA), ethylene and salicylic acid (SA)) were identified from the promoters of *ChCCOs*. Notably, the ABA-responsive element ABRE was found in promoters of all *ChCCOs*. The *ChNCED6* promoter contained the largest number of the ABA-responsive element ABRE (18 in total), followed by *ChNCED5* (8). Except for *ChCCD4*, the promoters of all other *ChCCOs* contained MeJA-related cis-acting elements (TGACG-motif and CGTCA-motif). The promoters of *ChCCD-like-a*, *ChCCD-like-b*, *ChCCD1* and *ChNCED6* contained auxin-responsive elements. The promoters of *ChCCD-like-a*, *ChCCD-like-b*, *ChCCD4*, *ChNCED1* and *ChNCED6* contained GA-responsive elements. The promoters of *ChCCD4*, *ChCCD7*, *ChCCD8* and *ChNCED6* contained the ethylene-responsive element ERE, and the *ChCCD4*, *ChNCED1* and *ChNCED5* promoters contained the SA-responsive TCA-element.

Among the stress-responsive elements, the defense and stress-related element MYB was identified in the promoters of all *ChCCOs*. The *ChNCED5* promoter contained the largest number of MYB elements (a total of 7). However, except for *ChNCED5*, all the promoters of other *ChCCOs* contained the anaerobic inducible element ARE. Except for *ChNCED6*, all the promoters of *ChCCOs* contained the high-temperature response element STRE. Except for *ChNCED1* and *ChNCED6*, the promoters of all other *ChCCOs* contained drought-inducibility-related elements. Moreover, the low-temperature responsive element LTRS was found in the promoters of *ChCCD-like-a*, *ChCCD1* and *ChCCD8*.

Distributions of transcription factor binding sites (TFBS) on *ChCCOs’* promoters were also analyzed. Totally, binding sites for 43 TFs were identified in the *ChCCOs’* promoters (Figure S5). Among them, the total number of ERF binding sites identified in promoters of *ChCCOs* was the largest (584), followed by bHLH (410), Dof (396) and BBR-BPC (378). In the promoters of *ChCCD1*, *ChCCD8*, *ChNCED1* and *ChCCD7*, binding sites for ERFs were found to be the most abundant, accounting for 127, 127, 126 and 103, respectively. The *ChCCD7* promoter contained 103 binding sites for BCR-BPCs. In the promoter of *ChCCD4*, the binding site for TCP was the most abundant (113). In the promoter of *ChCCD-like-a*, the binding site for MYB was the largest (45). The *ChCCD-like-b* promoter contained 45 Dof binding sites and 44 MYB binding sites. The bHLH binding sites were the most abundant in the promoters of *ChNCED6* (168) and *ChNCED5* (73), respectively.

### 2.5. Protein–Protein Interaction Analysis of ChCCOs

**Figure 2:**
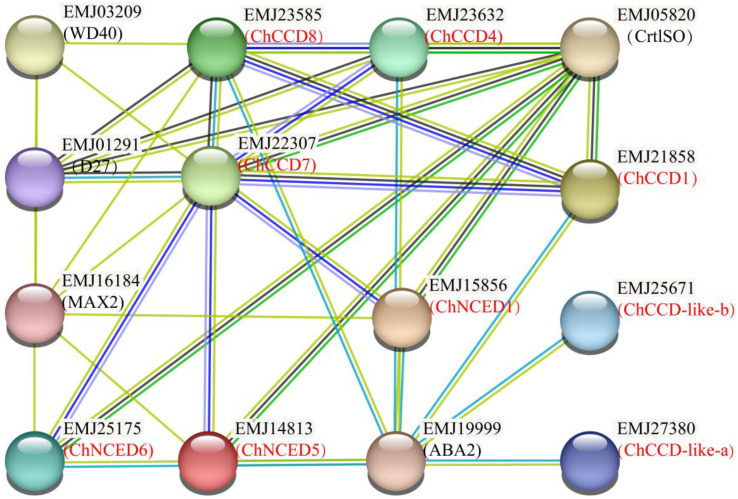
Protein–protein interaction network for ChCCOs based on the *Prunus persica* protein database.

Based on the *P*. *persica* protein database, possible interacting proteins of ChCCOs were predicted. Results showed that all the ChCCOs were homologous proteins of *P. persica* CCOs (Figure 2). ChCCD-like-a, ChCCD-like-b, ChCCD1, ChCCD8, ChNCED1 and ChNCED5 were predicted to have the ability to interact with ABA2. ChCCD7 and ChCCD8 could interact with WD40, D27 (DWARF27) and MAX2 (MORE AXILLARY BRANCHING2). In addition, all the three ChNCEDs were predicted to be interacting proteins of MAX2. Moreover, ChCCD1, ChCCD4, ChCCD7 and all the three ChNCEDs were predicted to have the ability of interacting with CrtlSO.

### 2.6. Gene Expression Analysis of ChCCOs

**Figure 3:**
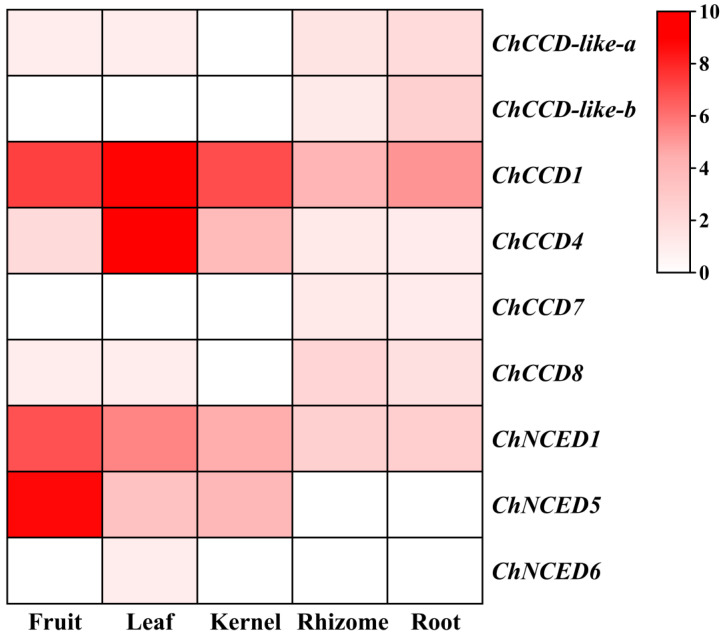
Heatmap for the transcriptome data-based expression analysis of *ChCCOs* in the fruit, leaf, kernel, rhizome and root of *C. humilis*. For heatmap drawing, log_2_(FPKM + 1) values of *ChCCO* genes were used. The redder the color, the higher the gene’s expression, and white represents no expression.

According to the transcriptome data of five *C. humilis* organs, including fruit, leaf, kernel, rhizome and root, we found that the expression levels of *ChCCOs* in different parts varied a lot (Figure 3). Of the nine *ChCCOs*, only *ChCCD1*, *ChCCD4* and *ChNCED1* expressed in all the five organs; *ChCCD-like-a* and *ChCCD8* showed no expression in the kernel, and its expression in fruit, leaf, rhizome and root were all relatively low (FPKM < 2); *ChNCED5* showed expression in fruit, leaf and kernel; *ChCCD-like-b* and *ChCCD7* expressed in only rhizome and root; and the expression of *ChNCED6* was leaf-specific.

Among the six fruit-expressing *ChCCOs* (*ChNCED5*, *ChCCD1*, *ChNCED1*, *ChCCD4*, *ChCCD8* and *ChCCD-like-a*), *ChNCED5* expressed the highest (FPKM > 400), followed by *ChCCD1* and *ChNCED1* (both with FPKM > 100). The expression level of *ChCCD4* ranked the fourth (with FPKM about 2) among the fruit-expressing *ChCCOs*. Although *ChCCD8* and *ChCCD-like-a* showed expression in fruit, their expression levels were very low. There were seven *ChCCOs* expressed in the leaf of *C. humilis*. Among them, the expression of *ChCCD1* and *ChCCD4* ranked top two, and their FPKM values were both higher than 400. *ChNCED1* and *ChNCED5* also expressed relatively high in the leaf. However, the expression levels of the other three leaf-expressing *ChCCOs* (*ChCCD-like-a*, *ChCCD8* and *ChNCED6*) were all very low (FPKM < 0.1). The expression levels of the four *ChCCOs* expressing in the kernel followed the order of *ChCCD1* > *ChNCED1* > *ChNCED5* > *ChCCD4*. And the FPKM value of *ChCCD1* in the kernel was more than 100. Except for *ChNCED5* and *ChNCED6*, all other *ChCCOs* showed expression in *C. humilis* rhizome and root with *ChCCD1* and *ChNCED1* both ranking top two.

The expression of the same *ChCCO* gene also showed obvious spatial differences. For example, *ChCCD1* expressed the highest in the leaf, followed by in the fruit and kernel; *ChCCD4* expressed the highest in the leaf, followed by in the kernel and fruit; the expression levels of *ChNCED1* and *ChNCED5* in the fruit were much higher than that in the other four organs.

**Figure 4:**
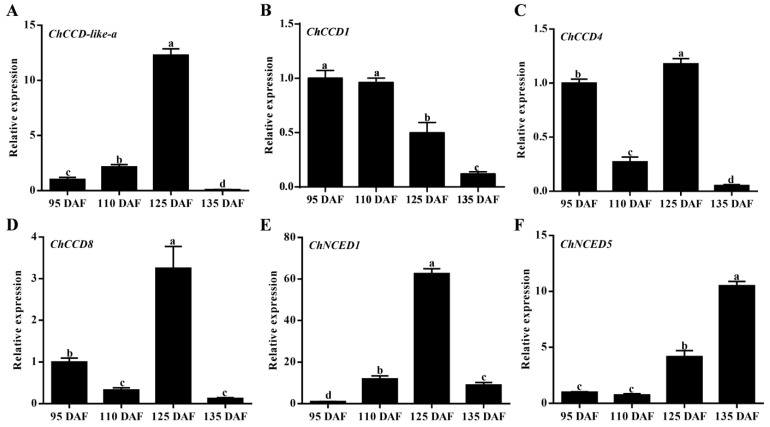
Quantitative real-time PCR analysis results of *ChCCOs* in fruits at four different ripening stages. (**A**–**F**) represents expression analysis result for *ChCCD-like-a*, *ChCCD1*, *ChCCD4*, *ChCCD8*, *ChNCED1* and *ChNCED5*, respectively. DAF: days after flowering. The different letters above the columns represent significant differences at *p* < 0.05 level.

The *C. humilis* fruits are rich in carotenoids and carotenoid-derived compounds [27]. To analyze the expression patterns of fruit-expressing *ChCCO* genes in fruits at different ripening stages, quantitative real-time PCR (qRT-PCR) analysis was performed (Figure 4). Results showed that the expression levels of *ChCCD-like-a* and *ChNCED1* increased sharply at 125 DAF (the color turning stage) but decreased at 135 DAF (the maturity stage). *ChCCD4* and *ChCCD8* exhibited a ‘fall-rise-fall’ expression change pattern during fruit ripening, and their expression levels both peaked at 125 DAF, followed by that in the fruit at 95 DAF. The expression of *ChCCD1* in fruits at 95 DAF and 110 DAF were significantly higher than that in fruits at 125 DAF and 135 DAF, and its expression at 135 DAF was found to be the lowest. However, the expression of *ChNCED5* in fruits at 125 DAF and 135 DAF was significantly higher than that in fruits at 95 DAF and 110 DAF, and its expression increased as fruit ripened. Except *ChCCD1* and *ChNCED5*, the relative expression levels of other fruit-expressing *ChCCOs* were all the highest at 125 DAF. Moreover, the expression levels of all the fruit-expressing *ChCCDs* (including *ChCCD-like-a*, *ChCCD1*, *ChCCD4* and *ChCCD8*) were the lowest at 135 DAF.

### 2.7. Prokaryotic Expression and Enzyme Assay Analysis of ChCCD1 and ChCCD4 Proteins

The contributions of CCD1 and CCD4 in carotenoid degradation and apocarotenoid accumulation have been frequently demonstrated in many plant species [14,15]. To clarify the functions of ChCCD1 and ChCCD4, pET-ChCCD1 and pET-ChCCD4 prokaryotic expression vectors were constructed and individually transformed into *E. coli* BL21(DE3). After protein expression activation using IPTG, SDS-PAGE gel electrophoresis was used to detect the expression of ChCCD1 and ChCCD4 proteins. Results showed that *E. coli* BL21(DE3) carrying pET-ChCCD1 and pET-ChCCD4 could, respectively, express recombinant proteins with a molecular weight of about 61 kD and 65 kD (Figure S6), indicating that ChCCD1 and ChCCD4 proteins were correctly expressed.

**Figure 5:**
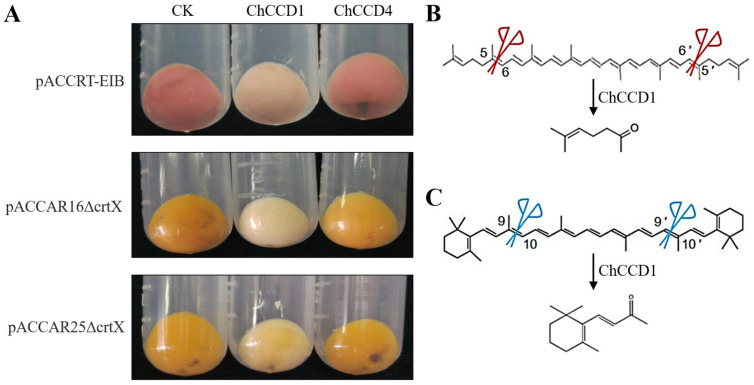
Functional analysis results of ChCCD1 and ChCCD4 proteins. (**A**) The influences of ChCCD1 and ChCCD4 expression on the color changes of *E. coli* strains that can accumulate lycopene (carrying pACCRT-EIB vector), β-carotene (carrying pACCAR16ΔcrtX vector) and zeaxanthin (carrying pACCAR25ΔcrtX vector); CK: control bacteria with no IPTG addition. (**B**) ChCCD1 can cleave lycopene into 6-methyl-5-heptene-2-one. (**C**) ChCCD1 can cleave β-carotene into β-ionone.

To reveal the roles of the ChCCD1 and ChCCD4 in carotenoid degradation and apocarotenoid accumulation, pET-ChCCD1/pET-ChCCD4 vectors were introduced into *E. coli* BL21(DE3) together with plasmid pACCRT-EIB/pACCAR16ΔcrtX/pACCAR25ΔcrtX. Before IPTG addition, the color of bacterial cultures carrying pET-ChCCD1/ChCCD4 and pACCRT-EIB, pET-ChCCD1/ChCCD4 and pACCAR16ΔcrtX and pET-ChCCD1/ChCCD4 and pACCAR25ΔcrtX was red, yellow-orange and yellow-orange (Figure 5), respectively. After IPTG induction, bacterial cultures expressing recombinant ChCCD1 exhibited remarkable color changes, varying from a lighter color (cultures expressing pET-ChCCD1 and pACCRT-EIB/pACCAR25ΔcrtX) to almost white (cultures expressing pET-ChCCD1 and pACCAR16ΔcrtX). However, the ChCCD4 expression did not result in significant bacterial culture color change. These results indicated that ChCCD1 could cleave lycopene, β-carotene and zeaxanthin. ChCCD4, however, could not cleave these substrates as efficiently as ChCCD1.

**Figure 6:**
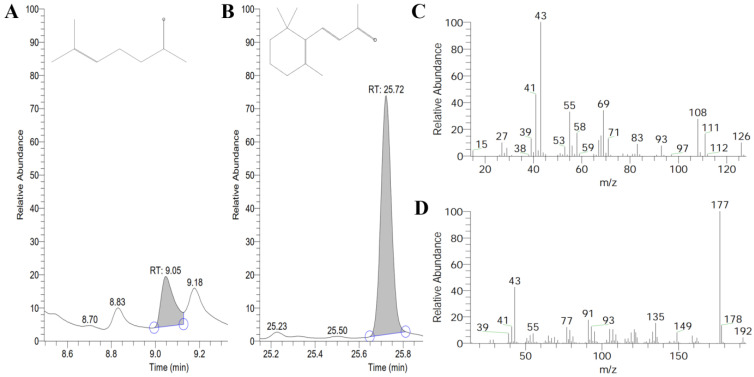
GC-MS detection results of the ChCCD1 cleaved volatile products of lycopene and β-carotene in *E. coli.* (**A**,**B**) for the cleaved volatile products of lycopene and β-carotene, respectively; (**C**,**D**) fragments pattern for 6-methy-5-hepten-2-one and β-ionone, respectively. Blue circles in (**A**) and (**B**) represent starting and ending time points of peak.

The volatile products of ChCCD1 and ChCCD4 in *E. coli* strains that can accumulate lycopene, β-carotene and zeaxanthin were further determined using headspace gas chromatography/mass spectrometer. Results showed that, after IPTG induction, among the volatiles released by the bacterial culture expressing pET-ChCCD1 and pACCRT-EIB, 6-methyl-5-heptene-2-one was detected (Figure 6). This indicated that ChCCD1 can cleave lycopene at 5, 6 and 5′, 6′ positions to produce 6-methyl-5-heptene-2-one. In the volatiles released by the bacterial culture expressing pET-ChCCD1 and pACCAR16ΔcrtX, β-ionone, an aroma substance produced by oxidative cleavage of β-carotene, was detected (Figure 6). This indicated that ChCCD1 can cleave β-carotene at 9, 10 and 9′, 10′ positions to produce β-ionone. No cleaved carotenoid products were detected in the volatiles released by other bacterial cultures.

## 3. Discussion

In this study, for the first time, we performed whole genome-wide identification and characterization of the *CCO* genes in *C. humilis*. Totally, nine *ChCCOs* (including six *ChCCDs* and three *ChNCEDs*) belonging to six subfamilies were obtained. This classification was supported by their gene structures and conserved motifs in their encoded proteins. Most of the ChCCOs contained four histidine active sites, which might be closely related to their iron-binding abilities [28,29]. Subcellular localization analysis revealed that ChCCOs were mainly localized in cytoplasm and chloroplast, which was consistent with the CCOs from many other plant species [13,30,31,32]. Synteny analysis revealed that *ChCCD-like-a* and *ChCCD-like-b* were tandem duplicated genes, suggesting that the tandem duplication of CCD-like subfamily members contributed to the amplification of the *CCO* gene family in *C. humilis*.

Accumulated evidence demonstrated that *CCOs* might be involved in the plant responses to phytohormones and abiotic stresses [33,34,35,36]. In this study, we identified many phytohormone- and stress-responsive elements in the promoters of *ChCCOs*. The ABA-responsive element identified in the promoters of *CCD* genes from six Cucurbitaceae species was reported to be the most abundant among all the phytohormone-responsive elements [37]. Similarly, the abundance of an ABA-responsive element in the promoters of the litchi CCD1, CCD4, CCD7, CCD-like and NCED subfamily genes ranked the first among all the phytohormone-responsive elements [38]. Consistently, in our study, all the promoters of *ChCCOs* were predicted to contain the ABA-responsive element ABRE. There were nine, nine, nine and eight *ChCCOs* that contained MeJA-responsive elements, the anaerobic inducible element ARE, the high-temperature response element STRE and the drought-inducibility-related elements in their promoters, respectively. In addition, the promoters of *ChCCD-like-a*, *ChCCD1* and *ChCCD8* contained the low-temperature responsive element LTRS. These results suggested that *ChCCOs* might play roles in phytohormone and stress responses in *C. humilis*.

Transcription factors (TFs) play important roles in the biosynthesis of secondary metabolites including carotenoids. The expression of carotenoid metabolism-related genes has been continuously proven to be regulated by TFs [39,40]. In this study, we identified binding sites for 43 types of TFs in the promoters of *ChCCOs,* but the abundance of binding sites for different TFs varied a lot. For example, the binding site for ERF was the most abundant in the promoters of *ChCCD1*, *ChCCD7*, *ChCCD8* and *ChNCED1*; *ChCCD4* and *ChCCD-like-a* promoters had many binding sites for TCPs and MYBs, while *ChNCED6* and *ChNCED5* promoters were rich of bHLH binding sites. The distribution and abundance differences of TFBSs in their promoters suggested that the expression of *ChCCOs* might be regulated by different TFs.

The expression patterns of different *CCO* gene members varied a lot in different parts of plant species [3,37]. In this study, our transcriptome data-based gene expression analysis revealed that there were six, seven, four, seven and seven *ChCCOs* expressed in the fruit, leaf, kernel, rhizome and root, respectively. *ChCCD1*, *ChCCD4* and *ChNCED1* showed expression in all five organs, while the expression of *ChNCED6* was found to be leaf-specific. *ChCCD1* expressed much higher than other *ChCCOs* in the kernel, rhizome and root; *ChCCD1* and *ChCCD4* expressed highly in the leaf and *ChCCD1*, *ChNCED1* and *ChNCED5* showed high expression in the fruit. All this suggests that their roles in carotenoid degradation were spatially different in *C. humilis*. Moreover, our qRT-PCR analysis revealed that the expression patterns of *ChCCOs* in fruits at different ripening stages were also temporally different.

Our protein–protein interaction analysis also indicated that ChCCOs play different roles in *C. humilis*. Six ChCCOs (including ChCCD-like-a, ChCCD-like-b, ChCCD1, ChCCD8, ChNCED1 and ChNCED5) were predicted to interact with ABA2. In higher plants, ABA is derived from xanthophyll carotenoids via the C15 intermediate xanthoxin. ABA2, a xanthoxin dehydrogenase that catalyzes the conversion of xanthoxin to abscisic aldehyde, is a key protein function in ABA biosynthesis [41]. This indicates that these ChCCOs might play roles in ABA biosynthesis by interacting with ABA2. ChCCD7 and ChCCD8 were predicted to interact with WD40, D27 and MAX2. WD40 has been reported to function not only in the biosynthesis of flavonoids but also in the carotenoid-derived pigments [42]. D27 is a β-carotene isomerase that can catalyze the interconversion of all-trans- into 9-cis-β-carotene (the precursor of SLs) [43,44]. In saffron, CsD27-1 was found to be co-expressed with CCD7 and CCD8 in the mycorrhized roots [45]. MAX2 is a key regulatory gene in SL signal transduction [46]. The interactions of ChCCD7 and ChCCD8 with these proteins indicated that these two ChCCOs were involved in the carotenoid degradation and SL biosynthesis. All the three ChNCEDs were also predicted to be interacting proteins of MAX2, indicating that they also function in these processes. Additionally, ChCCD1, ChCCD4, ChCCD7 and the three ChNCEDs were identified to interact with CrtlSO, a carotenoid isomerase that can catalyze the cis-to-trans isomerization of poly-cis-isomer of lycopene into all-trans lycopenes [45], indicating again that these ChCCOs function in carotenoids degradation.

The roles of CCD1 in regulating the formation of apocarotenoid volatiles [17,18,19,20,31], and the function of the *CCD4* gene in regulating the cleavage of carotenoids [47], have been confirmed in many plant species. The *Rosa damascene* RdCCD1 was reported to have the ability to cleave a variety of carotenoids at the 9, 10 and 9′, 10′ positions to produce a C14 dialdehyde and two C13 products, and it could also cleave lycopene at the 5, 6 and 5′, 6′ positions to produce 6-methyl-5-hepten-2-one [15]. The melon CmCCD1 could cleave a variety of carotenoids at 9, 10 and 9′, 10′ positions to generate several kinds of apocarotenoids [48]. In rice, CCD1 has been reported to have the ability to convert lycopene into volatiles, pseudoionone, 6-methyl-5-hepten-2-one and geranial [49]. In this study, we functionally analyzed the cleavage ability of ChCCD1 and ChCCD4 on lycopene and β-carotene and zeaxanthin by co-expressing them together with genes that can induce carotenoid accumulation in *E. coli*. Results showed that ChCCD1 can oxidize lycopene at 5, 6 and 5′, 6′ positions to produce 6-methyl-5-heptene-2-one and can cleave β-carotene at 9, 10 and 9′, 10′ positions to produce β-ionone. These results suggest that ChCCD1 plays a key role in the β-carotene accumulation in *C. humilis* and contributes greatly to the β-carotene-rich characteristics of *C. humilis* fruits [26,27]. Although ChCCD1 expression can also lead to the degradation of zeaxanthin, no volatile products were detected through GC/MS. This can be explained by the fact that the cleaved products of zeaxanthin by ChCCD1 are not volatiles. In addition, we also investigated the function of ChCCD4 in carotenoid degradation and apocarotenoid biosynthesis. Results showed that it did not show an obvious influence on the bacterial culture color change and volatiles release, which might be related to its higher substrate specificity [22].

## 4. Materials and Methods

### 4.1. Plant Materials

The two-year-old *C. humilis* cv. ‘Jinou No. 1’ materials were collected from the *C. humilis* resource nursery of Juxin Demonstration Park at Shanxi Agricultural University. At 95, 110, 125 and 135 days after flowering (DAF), fruits used for gene expression analysis were harvested, washed three times with sterile water, quick-frozen in liquid nitrogen and stored in a −80 °C freezer for further use.

### 4.2. Identification of Cerasus humilis CCO Genes

The *C. humilis* genome file [26] was provided by Dr. Pengfei Zhang from Shanxi Agricultural University. The *Prunus persica* CCO protein sequences were downloaded from NCBI (https://www.ncbi.nlm.nih.gov/, accessed on 3 March 2023) and used as queries to BLASTP against the *C. humilis* protein data to screen ChCCOs under the criterion of e-value ≤ 1 × 10^−5^. Meanwhile, the RPE65 (retinal pigment epithelial membrane protein 65) domain Markov model (PF03055) downloaded from Pfam (http://pfam.xfam.org/, accessed on 3 March 2023) was used to search the putative CCO proteins in *C. humilis* using HMMER 3.0 under the criterion of e-value ≤ 1 × 10^−5^. As some annotated *ChCCOs* do not have complete sequences, unigenes that were annotated as *CCOs* in our transcriptome data were subjected to gene cloning and sequencing confirmation to obtain their full-length CDSs. The obtained candidate CCOs were further subjected to conserved domain confirmation, and only proteins containing the RPE65 domain remained.

### 4.3. Bioinformatic Analysis of ChCCOs and Their Encoded Proteins

The physiochemical properties, subcellular localization and the existence of chloroplast transit peptide in ChCCOs were analyzed using ProtParam (https://web.expasy.org/protparam/, accessed on 5 March 2023), CELLO (http://cello.life.nctu.edu.tw/, accessed on 5 March 2023) and ChloroP (http://www.cbs.dtu.dk/services/ChloroP/, accessed on 5 March 2023), respectively. For gene structure analysis of *ChCCOs*, TBtools [50] was used. MEME (http://meme-suite.org/tools/meme, accessed on 5 March 2023) was applied to analyze the conservative motifs in each member of ChCCOs (the motif number was set as 10, and other parameters were set as default values). All the *ChCCO* genes were mapped to *C. humilis* chromosomes according to their location information, and synteny analysis was performed using MCscanX (Multiple Collinearity Scan Toolkit X version). MEGA7 software (Molecular Evolutionary Genetics Analysis Version 7.0) was applied for the multiple sequence alignments of CCO proteins from *C. humilis*, *P. persica*, *Fragaria vesca*, *Solanum Lycopersicum*, *Oryza sativa* and *Arabidopsis thaliana* and for phylogenetic tree construction by using the Neighbor Joining (NJ) method with default parameters (bootstrap = 1000). TBtools was used to extract the 2000 bp sequences upstream from the start codons (ATG) of *ChCCOs* from the *C. humilis* genome data. Additionally, the extracted sequences were used as promoter sequences of *ChCCOs* and were subjected to PlantCARE (http://bioinformatics.psb.ugent.be/webtools/plantcare/html/, accessed on 6 March 2023) and PlantTFDB (http://plantregmap.gao-lab.org/binding_site_prediction.php, accessed on 6 March 2023) for the cis-acting element and transcription factor binding sites (TFBS) analysis, respectively. Given the close relationship between *C*. *humilis* and *P. persica*, the interacting proteins of ChCCOs were predicted using STRING (https://cn.string-db.org/, accessed on 7 March 2023) based on the *P. persica* protein database.

### 4.4. Gene Expression Analysis

Based on our transcriptome data, the expression patterns of *ChCCO* genes in the fruit, leaf, kernel, rhizome and root were analyzed. TBtools was used for drawing the heatmap of their expression levels.

Trizol (Invitrogen, CA, USA) and the PrimeScript RT Master Mix (Perfect Real Time) kit (Takara, Dalian, China) were used for isolating the total RNA from *C. humilis* fruits and for biosynthesizing the complementary DNA (cDNA) used for quantitative real-time PCR, respectively. Gene-specific primers used for quantitative real-time PCR were designed using Vector NTI (Table 2). The expression of *ChCCO* genes in fruits at different ripening stages was investigated on an ABI 7500 real-time PCR system. The amplification system contained 2 μL cDNA, 0.8 μL each of the forward and reverse primers (10 μM), 0.4 μL ROX Reference DyeⅡ, 10 μL SYBR solution and 6 μL ddH_2_O. The reaction procedure was set as follows: pre-denaturation at 95 °C for 3 min; denaturation at 95 °C for 15 s; annealing at 58.5 °C for 30 s; extension at 72 °C for 15 s; 40 cycles. Three biological replications were made for each gene. By using *ChActin* as an internal reference gene, the relative expression of *ChCCOs* in different samples was calculated using the 2^−ΔΔCT^ method.

### 4.5. Expression of ChCCD1 and ChCCD4 in Escherichia coli

To clone the full-length CDSs of *ChCCD1* and *ChCCD4*, gene-specific primer pairs with *Bam*HI digestion site sequences (GGATCC) in the forward primers and *Xho*I digestion site sequences (CTCGAG) in the reverse primers were designed (Table 2). Amplified PCR products were double digested with *Bam*HI and *Xho*I and introduced into the prokaryotic expression vector pET28, which had been digested using the same two enzymes to generate pET-ChCCD1 and pET-ChCCD4 vectors. Then, vectors were transformed into *E. coli* BL21(DE3) and incubated at 37 °C with gentle shaking at 125 rpm till OD_600_ of 0.5. The expression of recombinant ChCCD1 or ChCCD4 proteins was induced by the addition of isopropyl β-D-thiogalactopyranoside (IPTG, with a final concentration of 0.5 mM), after which the cultures were grown at 37 °C for an additional 5 h. SDS-PAGE gel electrophoresis was applied to detect the protein expression.

### 4.6. Enzyme Assays In Vitro and Volatile Compounds Detection

pET-ChCCD1/pET-ChCCD4 vectors were transformed into *E. coli* BL21(DE3) together with plasmids pACCRT-EIB (carrying *crtE*, *crtB* and *crtI* genes), pACCAR16ΔcrtX (carrying *crtE*, *crtB*, *crtI* and *crtY* genes) and pACCAR25ΔcrtX (carrying *crtE*, *crtB*, *crtI*, *crtY* and *crtZ* genes) [51], respectively. After color observation and PCR detection using gene cloning primers for vector construction (Table 2), positive colonies respectively carrying pET-ChCCD1 and pACCRT-EIB, pET-ChCCD1 and pACCAR16ΔcrtX, pET-ChCCD1 and pACCAR25ΔcrtX, pET-ChCCD4 and pACCRT-EIB, pET-ChCCD4 and pACCAR16ΔcrtX and pET-ChCCD4 and pACCAR25ΔcrtX, were inoculated into an LB liquid medium containing appropriate antibiotics and incubated at 37 °C till OD_600_ of 0.6. After IPTG addition, bacterial cultures were grown at 37 °C for an additional 5 h and gently shaken at 125 rpm to induce protein expression. Bacterial cultures that were not treated with IPTG were used as controls. The volatile compounds of bacterial cultures collected from the headspace were analyzed on a quadrupole GC/MS HP GCD (G1800A) coupled to an HP-5 silica capillary column (30 m × 0.25 mm) according to Huang et al. [14]. The oven temperature was held at 50 °C for 1 min and then increased to 200 °C at 4 °C/min intervals, with a helium flow rate of 1 mL/min. The EI-MS ionization voltage was 70 eV, and the ion source temperature was 280 °C. The mass range was recorded from 45 to 450 *m*/*z,* and spectra were evaluated with the Xcalibur software version 1.4.

## 5. Conclusions

In summary, for the first time, we identified and characterized the *CCO* gene family of *C. humilis*, investigated their expression patterns in different tissues and organs and in fruits at different ripening stages and functionally validated the roles of ChCCD1 in the degradation of lycopene, β-carotene and zeaxanthin. According to the results obtained in our study, it can be concluded that ChCCD1 plays a key role in carotenoid degradation and apocarotenoid accumulation in *C. humilis*.

## Figures and Tables

**Table 1:** Physiochemical prosperities and subcellular location analysis results of ChCCOs. Chr: chromosome; CDS: coding sequence; bp: base pair; Chr: chromosome; AA: amino acid; pI: isoelectric point; GRAVY: grand average of hydropathicity.

Gene Name	Chr	CDS/bp	Number of AA	Molecular Weight/Da	pI	Instability Index	GRAVY	Subcellular Location
*ChCCD-like-a*	Chr1	2109	702	78,364.26	5.44	36.82	−0.196	Chloroplast, Cytoplasm
*ChCCD-like-b*	Chr1	1677	558	62,470.54	5.49	28.40	−0.292	Cytoplasm
*ChCCD1*	Chr5	1644	547	61,777.14	6.05	34.76	−0.258	Cytoplasm
*ChCCD4*	Chr1	1794	597	65,717.9	6.10	38.51	−0.229	Chloroplast, Cytoplasm
*ChCCD7*	Chr5	1848	615	68,499.76	5.67	46.35	−0.219	Cytoplasm
*ChCCD8*	Chr1	1698	565	62,461.97	6.20	34.98	−0.270	Cytoplasm
*ChNCED1*	Chr3	1899	632	70,288.74	6.73	45.34	−0.435	Chloroplast, Cytoplasm, Nucleus
*ChNCED5*	Chr3	1854	617	68,027.19	6.45	46.67	−0.335	Cytoplasm
*ChNCED6*	Chr1	1818	605	66,921.84	6.97	38.59	−0.287	Chloroplast

**Table 2:** Information of primers used in this study. The nucleotide sequences underlined represent digestion site sequences of *Bam*HI (GGATCC) and *Xho*I (CTCGAG).

Gene Name	Forward Primer (5′–3′)	Reverse Primer (5′–3′)	Applications
*ChActin*	TTCAAAGACCAGCTCATCTGTGG	CAATGCCAGGGAACATAGTGGA	qRT-PCR
*ChCCD-like-a*	AAGTCAAGACCACCCTCTCCTCC	AACTCGTCTACGGGGCCAAAG	qRT-PCR
*ChCCD1*	ATGGCGGAGGTTGAAGATGAGG	TTGGTGGGAGGAGTTTCATCAAG	qRT-PCR
*ChCCD1*	CGGGATCCATGGCGGAGGTTGAAGATG	CCCTCGAGTTAGAGCTTTGCTTGTTCTTGC	Vector construction
*ChCCD4*	GGATGCCTTCTCTTCCTCTTTCC	CGCGAGCTTTTGTTGTTAGTGG	qRT-PCR
*ChCCD4*	CGGGATCCATGGATGCCTTCTCTTCCTC	CCCTCGAGCTACAACTTGTTGAGATCAC	Vector construction
*ChCCD8*	ATGGCTTCCATAGCATTTTCCG	AGCCACTATTGCCGCTCTCTCT	qRT-PCR
*ChNCED1*	CCTCTTCCTCTTCCAGCCCAA	GGCACTGGCTTTGAGGATTTAGA	qRT-PCR
*ChNCED5*	GCTCTTCCAAAAGCACCCAATT	GGAAGTGAAGAACCGAGGGAGAT	qRT-PCR

## Data Availability

All data is available in this article.

## Supplementary Materials

The following supporting information can be downloaded at: https://www.mdpi.com/article/10.3390/plants12112114/s1, Figure S1: Multiple sequence alignment results of ChCCOs. Red, yellow and green shading represent 100%, ≥75% and ≥50% amino acid sequence similarity, respectively; Figure S2: Chromosomal locations of *ChCCO* genes; Figure S3: Conserved motifs in ChCCOs proteins (A) and gene structures (B) of their corresponding genes; Figure S4: The identified cis-acting elements in *ChCCOs*’ promoters. Red, yellow and green colors represent the high, moderate and low abundance of cis-acting elements in promoters, respectively. Figure S5: Heatmap for the transcription factor binding sites identified in promoters of *ChCCOs*. Red, yellow and green colors represent the high, moderate and low abundance of TFBS in promoters, respectively. Figure S6: SDS-PAGE gel electrophoresis detection results of ChCCD1 and ChCCD4 proteins. M: protein marker; 1–3: *E. coli* strains expressing ChCCD1; 4 and 5: *E. coli* strains carrying pET-ChCCD1 without IPTG induction; 6: *E. coli* strains carrying pET-ChCCD4 without IPTG induction; 7–9: *E. coli* strains expressing ChCCD4. Arrows represent target bands for recombinant ChCCD1 and ChCCD4.
