# Supplementary material for: Characterization of Carotenoid Cleavage Oxygenase Genes in Cerasus humilis and Functional Analysis of ChCCD1
Source: Plants (Basel). 2023 May 26;12(11):2114. doi: 10.3390/plants12112114 (PMC10255781; doi:10.3390/plants12112114)
Supplement: Supplementary file 1 [file plants-12-02114-s001.zip › Figure S1.pdf]

```
ChCCD-like-a   ..................................................................MYVNSTEVLCSSEEVKTTLSSSAFMPLLMRKVEQV   35
ChCCD-like-b   ....................................................................................................   0
ChCCD1         ........................................................................................MAEVEDEEKQQIK   13
ChCCD4         .......................MDAFSSSFLSTFPTQNLSLSPAIATPKLSISSVRIEERPSSPPPASKPTSTKVPQPPKTPSLPLTTKARDYNNDSTF   77
ChCCD7         .........................................................MQANPNPFQIIPKQIFPSLIKLPPVHQSPSPVPTKPPRAISIS   43
ChCCD8         ...................................................................MASIAFSGTSGNSFFLPTASVSDYSNQNIIDGF   33
ChNCED1        .MPSTAAATTGTWVKPVTTRRHYLSCTNSSNLATNQLGFPPSSSSSPIFFSRKPIHRKSTPNTLTCSLQAPSLLQIPKHTPSYQQPSSSSSTTIFTPSTT   99
ChNCED5        MAALPKAPNSNSWATSTAQMPHTLLSSSSSSLV.DPMGFPKRSISLRKTHPKAATRRTTS...IHCALQSPSVLHFPNQ..PYNQPIITKEATSPKPNNS   94
ChNCED6        ...........................MHASLHFTTINTPPSSSTLPKNPIQITRSPTTQ..ITCKILTNPSKKTLPQKTQKPLWPTLPPPNTKPLLPQV   71

ChCCD-like-a   LPMQINVS..............KTFKNTSSKVLDFLVDSMFEF.........VDQSLLPSQSNFGPVDELGGEVVITSSIRGKIPDDFPDGVYIRNGPNP   112
ChCCD-like-b   ........................MKNTSGKILDALVNSMFKF.........VDQPLLPSQKNFAPVEEIGSLVEINCT.EGEIPPDFAEGVYIRNGNNP   66
ChCCD1         SSGIVVPN..............PKPNKGFASKVVDLIENLIVK.....LMYDPSQPHHYLAGNFAPVL..DETPPTNNLPVIGSLPECLNGEFVRVGPNP   92
ChCCD4         SAAKKQTD..............PTLPAVIFNALDDIINNFIDP.....PLRPSVDPKHVLSNNFAPV...DELPPTECEIIQGSLPPCLDGAYIRNGPNP   155
ChCCD7         TPNETDRG...............PITISTLDVVDDSVAAFWDYQFLFVSQRSEQTEPISLRVVDGSVP..SDFPSGTYYLTGPGLFADDHGSTVHPLDGH   126
ChCCD8         NPLKKNLS............DTRKKHNGRGLVSTNVASPPLPAVAPLLPRRESGNSGYRSHVAWTSV...RQERWEGELLVQGEIPLWLKGTYLRNGPGL   118
ChNCED1        SNPKENKTQPSSKSSKPVPQQWNFLQRAAAMALDAMESALLSK.EQQHPLPKTADPKVQIAGNFAPV...PEHPVEHSLPVTGKIPASIQGVYVRNGANP   195
ChNCED5        THHHHQ.............PPQWNLLQKAAAMAIDMVEGALVSR.ERQNPLPKTSDPRVQIAGNYAPV...PEQPVHHSLPITGTIPECINGVYVRNGANP   178
ChNCED6        NPIKPNIH..........QPRLNPLQKLAASLLNTIETSLIEPLEKKQSLPKTIDPAVQISGNFAPV...RECPVQYCLEVMGQIPDCLRGVYVRNGANP   158

ChCCD-like-a   LFGGLKSTKSIFGRSSHIWIEGEGMLHALYFSKDVSDGCWTVRYNNRHVETETFKLEKQ.KNKPSFLPAIGGDS..PAILSAYLLNLLRFGKVNKYLSNT   209
ChCCD-like-b   LFGGLKSAVSIFGQSHHSWVDGEGMLHAVYFKKD.QNGSWIISYKNRYVETETFKLEKHIHNKPCFLPVLQGDA..SAVLAALLLNWLRFGAVNKYFSNT   163
ChCCD1         KFAPV.........AGYHWFDGDGMVHGMRIKDGK.AMYVSRYVRTSRLKQEEYFGGAKFMKIGDLKGLFGLLMVNMQILRAKLKVLDMSYGNGTG..NT   180
ChCCD4         QYLPR.........GPYHLFDGDGMLHSVRISKGR.AVLCSRYVKTYKYTIERDAGYPLLPNVFSGFNGLTASATRGALSAARVFTGQYNPANGIGLANT   245
ChCCD7         GYLRA...FKLDGFGEDVKFMAKYVKTEAQVEEHDPLTDTWRFTHRGPFSVLKGGQKVGNTKVMKNVANTSVLRWGQRLLCLWEGGDPYEIESETLDTIG   223
ChCCD8         WHIGD........YNFRHLFDGYAMLVKLQFEDGR......LIAGHRQIESEAYKAAMKTQKLCYREFSEVPKPDNFLSYIGELANLFSGASLTDNANTG   204
ChNCED1        LHEPV.........AGHHFFDGDGMVHALQFKDGA.ASYACRFTETHRFNQERDLGRPLFPKAIGELHGHSGIA.RLLLFYARGMFGLVDPTHGIGVANA   284
ChNCED5        LFEPV.........AGHHLFDGDGMVHAVTIDSGS.ASYACRFTETQRLVQEREFGRPVFPKAIGELHGHSGIA.RLLLFYARGVLGLVDKNHGTGVANA   267
ChNCED6        MFAPA.........GGHHLFDGDGMIHALTLGSANRASYSCRYTRTSRLEQEAKMGRSIFPKPIGELHGHSGLA.RLCLFMARAAVGLVDCSRGTGVANA   248

ChCCD-like-a   NVFEHSGKFYSIAENHIPQEIDIFT...LETLGNWDVSGAWNRPFTSHPKRAPGTGELVILGIDAVK...PFMEIGVVSADGKELIHKVDL.NLDRCSLC   302
ChCCD-like-b   NVFEHSGRVYSVTENYLPQEVDIST...LETLSDWDVNGAWDRPFTGHPKKAPSSGELVIMGTDAKK...PYYVLGVISADGKKL.HKADL.KFKRSVLS   255
ChCCD1         ALIYHHGKLLALSEADKPYVLKVLEDGDLQTVGLLDYDKRLTHSFTAHPKVDPFTGEMFTFGYSHTP...PYITYRVISKDGFMHDPIPI..TVADPIMM   275
ChCCD4         SLAFFGNQLYALGESDLPYSLRLTSNGDIQTLGRHDFDGKLFMSMTAHPKIDPETGEAFAFRYGPLP...PFLTYFRFDANGTKQPDVPIF.SMVTPSFL   341
ChCCD7         SIG.LMDGCDSGVESRDHGGSVWDVAARLLKPILYGIFKMPPKRLLSHYKLDARRSRLLMVSCNAEDMLLPCSNFTFYEYDSNFKVLGKQEFNIPDHLMI   322
ChCCD8         VVMLADGRVVCLTETQKGSIVIDPAT.LDTLGKFEYSDTLGGLIHSAHPIVTETEFLSLLPDLINPG...YLAVRMEPGTNERKVIGRVDCCRGPAPGWV   300
ChNCED1        GLVYFNGRLLAMSEDDLPYHVKIAETGDLKTVGRYDFDKQLKSTMIAHPKVDPTTGELFALSYDVVQR..PYLKYFKFSPDGEKSPDVEI..NLDQPTMM   380
ChNCED5        GLVYHNGRLLAMSEDDLPYQVRVTKSGDLETVGRYDFNSQLGSTMIAHPKVDPESGSLFALSYDVVQK..PYLKYFQVSPDGAKSPDVEI..PLAGPTMM   363
ChNCED6        GLVYFNGRLLAMSEDDLPYNVKIKGDGDLETIGRFDFNGGLDRPIIAHPKVDPVTGELHVLSYDVVKK..PYLKYFKFGASGTKPRDVDI..TLDQPTMV   344

ChCCD-like-a   HELGVTQRYNAIMDFPLTLDINRLVN........GGPLVKYNQEGYARIGVMPRFG.DANSIHWFIVESH...CTFHIINSFEDGDEVVVWGCKALDSVI   390
ChCCD-like-b   HDIGVTQKYNVIIDHPLTVDIQRLAM........GGQLMKYEKEGFARIGVMPRYG.DAESVKWFEVQTS...CTFHILNCFEEGDEVVVRGCRALTSLI   343
ChCCD1         HDFAITENYAIFMDLPLYFRPKEMVKE.......KKLIFTFDPTKKARFGVLPRYAKDDLLIRWFELPNC...FIFHNANAWEEEDEIVLITCRLENPDL   365
ChCCD4         HDFAITKKYAIFVDIQIGMNPIDMITK.......GASPVGLDPSKVSRIGVIPRYAKDETEMRWFDVPGF...NIIHAINAWDEEDAIVMVAPNILSAEH   431
ChCCD7         HDWAFTDTHYILFANRIKLDVVGAMTAVCGTSPMITALSVNPSKATSPIYLLPRSPNENGRDWRVPIEASSQLWLLHVANAYENLDENGNLEIEIHAS..   420
ChCCD8         HSFPVTEHYIIAPEMPLRYCAGNLLKAE......PTPLYKFEWHPESKAFMHVMCKASGNIVASVEVPLY...ITFHFINAYEEKDEDGRVTAVIADCCE   391
ChNCED1        HDFAITERYVVIPDQQVVFKLQEMIT........GGSPVIYDKNKMSRFGILDKNAKDASGIRWVDCPDC...FCFHLWNAWEEPETDD...VVVIGSCM   466
ChNCED5        HDFAITENYVVIPDQQVVFKLQEMIT........GGSPVIYDKDKMSRFGILKKNAKNADDLVWVDSPDT...FCFHLWNAWEEPQSDE...VVVIGSCM   449
ChNCED6        HDFAITQNYVVIPDQQVVFKLSEMIK........GGSPVIYDPNKTSRFGILPKNDVDESGIQWIEVPNC...FCFHLCNAWEETSEVGDPIIVVIGSCM   433

ChCCD-like-a   PGPDMSL...NQFGWLPRRFKPADPSKE..NNDDISAEDGKLFSHAYEWRLNMNTGKVMERYLTGK..EFSMDFPMINGAFSGVKNRYGYTQVVDSIASS   483
ChCCD-like-b   PGPDDDVGFNNKFEWFSKGFNFAHHTKDVSSADDIFAEPGYFFSCVYEWRLNMVSGEVEEKNLTGT..EFSMEFPFINDQVTGLKHKYGYTQVVDSKASS   441
ChCCD1         DMVNGPV...........................KEKLEN.FKNELYEMRFNLKTGLASQKKLSES....AVDFPRVNESYTGRKQRYVYGTLLDSIA..   431
ChCCD4         TL................................ERMDL..IHASVEKVRIDLKTGIVSRQPISTR....NLDFAVFNPAYVGKKNKYVYAAVGDPMP.   491
ChCCD7         .........................................ACSYEWFNFQKLFGYDWQSGKLDPSIMNINGSQSKTLPHLIQVSINLDVSG......   471
ChCCD8         HNADTTI..LDKLRIHNLRSYTGQDVLPDARVGRFRIPFDGSAYGKLEAALDPNEHGRGLDMCSINPAYLGKQYRYAYACGAQRPCN......FPNTLTK   483
ChNCED1        TPPDSIF...........................NECDEK.LESVLSEIRLNLKTGKSTRRRICAE..NVNLEAGMVNRNKLGRKTRFAYLALAEPWP..   534
ChNCED5        TPPDSIF...........................NECDES.LKSVLSEIRLNLKTGESTRRAILSESEHVNLEAGMVNRSRLGRKTRFAYLAIAEPWP..   519
ChNCED6        DPPDSVF...........................NEHENNPIRAELTEMRMNLRTQESTRRVLVPA...LNLEVGQVNKEVVGRKSKYVYMAIAEPWP..   501

ChCCD-like-a   TSGMLKYGGLAKLHFEEPADVSL...RNESQLEVETHTFEEKSFCSGAAFVPKQGG.......LEEDDGWAITFVHNEDTNISQVYVIDTKKFSDEPVAK   573
ChCCD-like-b   TCGMGKFGSLAKLYLEESYATTSAEGKCEELIKVEYHKFEENNFCNGSVFVARHGGKG.....MEEDDGWIVTFVHNEETDVTQVHVIDASRFGS.....   531
ChCCD1         .....KVTGVVKFDLHAAPELGK.TKIEVGGNVQGLYDLGPGRFGSEAIFVPRVP.GVTSE...EDDGYLIFFVHDEKTGKSSIHVIDAKKMSS.....   515
ChCCD4         ......KISGVVKLDV.........SNVEHKECIVASRMFGPGCYGGEPFFVAREPENPEAD..EDDGYVVTYVHDEKAGESRFLVMDAKSPRL.....   568
ChCCD7         ...........................................................SCQKCDVEPLNQWNKSSDFPVINPAFSGSKN.........   502
ChCCD8         IDL.....VNKKAKNWHDEGAVPSEPFFVARPGATEEDDGVVISMISGKNGDGYALLLDGSTFQEIA.................................   545
ChNCED1        .....KVSGFAKVDL..............STGEVKKHIYGDEKYGGEPLFLPRDQNS......ENEDDGYILAFVHDEKEWKSELQIVNAMDLKVE....   605
ChNCED5        .....KVSGFAKVDV..............STGEVKKFIYGDKKYGGEPFFVPNTELG......SSEDDGYIMTFVHDEKTWKSELQIVNAVTLKLE....   590
ChNCED6        .....RCSGIAKVDL...............ETGSVTKHLYGTGRFGGEPFYVPTRKNDSMGNHVEEDDDGFIMGFVRDEVDESSELVILKASSMKQV...   578

ChCCD-like-a   ITLPCRMQQLLAVPSCALQVNGSFQTHFISESFNHLKTSLSSAAKPLFKELQQLPIKKVNNVSETMKNTSGKILDALVNSMFKFVDQPLLPSQKNFAPVE   673
ChCCD-like-b   ..........EAIAKLTLPQRVPYGFHGTFVSMPSQS..................................................................   558
ChCCD1         ..........DPVAVVELPHRVPYGFHAFFVTEEQLQEQAKL.............................................................   547
ChCCD4         ..........DIVADVRLPRRVPYGFHGLFVKESDLNKL................................................................   597
ChCCD7         ..........TYIYAAASSGSRSALPHFPFDMVAKLNVSTKSVLTWSVGSRRFIGEPTFIPKGS........EEDDGYILVVEYAVSVQRCYLVILDSK   583
ChCCD8         ..............RAKFPYGLPYGLHGCWVPSK.....................................................................   565
ChNCED1        .............ATIQLPSRVPYGFHGTFISSKDLEKQA...............................................................   632
ChNCED5        .............ATVKLPSRVPYGFHGTFIESKDLANQA...............................................................   617
ChNCED6        .............GLVRLPARVPYGFHGTFISEQDLKEQA...............................................................   605

ChCCD-like-a   EIGSLVEINCTEGEIPLILLRVTIHYLEV.......................................................................   702
ChCCD-like-b   ....................................................................................................   558
ChCCD1         ....................................................................................................   547
ChCCD4         ....................................................................................................   597
ChCCD7         RIGGEDALVARLEVPKHLNFPLGFHGFWATAE....................................................................   615
ChCCD8         ....................................................................................................   565
ChNCED1        ....................................................................................................   632
ChNCED5        ....................................................................................................   617
ChNCED6        ....................................................................................................   605
```
