# Supplementary material for: Characterization of Carotenoid Cleavage Oxygenase Genes in Cerasus humilis and Functional Analysis of ChCCD1
Source: Plants (Basel). 2023 May 26;12(11):2114. doi: 10.3390/plants12112114 (PMC10255781; doi:10.3390/plants12112114)
Supplement: Supplementary file 1 [file plants-12-02114-s001.zip › Figure S4.pdf]

Function		Motif	*ChCCD-like-a*	*ChCCD-like-b*	*ChCCD1*	*ChCCD4*	*ChCCD7*	*ChCCD8*	*ChNCED1*	*ChNCED5*	*ChNCED6*
Light responsive	Light	chs-CMA1a	1	0	0	0	1	0	0	0	0
		G-box	1	2	2	0	8	2	6	3	12
		TCCC-motif	1	0	0	2	1	0	0	0	1
		AE-box	1	1	0	0	0	0	0	0	1
		Gap-box	1	0	0	0	0	0	0	0	0
		GT1-motif	0	3	2	2	0	1	1	1	0
		I-box	0	1	0	1	0	0	0	2	1
		ACE	0	1	0	0	0	0	1	0	2
		GATA-motif	0	0	2	1	2	0	1	0	3
		Box 4	0	0	2	4	1	6	2	8	3
		TCT-motif	0	0	2	0	0	0	1	3	1
		GA-motif	0	0	0	2	0	0	1	0	0
		MRE	0	0	0	1	0	1	0	0	2
		3-AF1 binding site	0	0	0	0	1	0	0	0	0
		LAMP-element	0	0	0	0	1	0	0	0	0
		RY-element	0	0	0	0	0	1	2	0	0
		Box II	0	0	0	0	0	0	0	1	1
		chs-CMA2a	0	0	0	0	0	0	0	1	1
Growth and development	Meristem expression	CAT-box	2	0	3	0	1	1	0	0	0
	Circadian control	circadian	1	0	0	0	0	0	0	0	1
	Zein metabolism	O2-site	0	2	1	0	3	1	0	3	1
	Endosperm expression	GCN4 motif	0	0	0	0	0	0	1	0	0
Phytohormone responsive	Abscisic acid	ABRE	2	2	4	1	5	4	5	8	18
	Auxin	AuxRR-core	0	1	0	0	0	0	0	0	0
		TGA-element	0	0	2	0	0	0	0	0	1
		TGA-box	1	0	1	0	0	0	0	0	0
	MeJA	TGACG-motif	3	3	5	0	1	3	1	1	3
		CGTCA-motif	3	3	5	0	1	3	1	1	3
	Gibberellin	P-box	3	1	0	0	0	0	0	0	1
		TATC-box	0	0	0	1	0	0	1	0	0
	Ethylene	ERE	0	0	0	2	2	1	0	0	2
	Salicylic acid	TCA	0	0	0	1	0	0	2	1	0
Stress responsive	Defense and stress	Myb-binding site	1	1	1	0	0	0	0	0	0
		MYB	6	4	5	3	2	1	1	7	2
		MYB recognition site	2	2	0	0	0	1	0	0	0
		CCAAT-box	2	2	0	0	0	1	0	0	0
		TC-rich repeats	0	1	1	0	0	0	0	0	0
		W box	0	0	2	2	0	0	1	1	2
		DRE core	0	0	0	0	1	0	0	0	0
	Wound	WRE3	1	0	1	6	0	1	1	4	1
		WUN-motif	1	2	0	1	1	0	0	1	1
	Anaerobic induction	ARE	1	2	4	2	2	1	4	0	6
	High-temperature	STRE	2	2	6	5	2	2	1	1	0
	Low-temperature	LTR	1	0	1	0	0	1	0	0	0
	Drought-inducibility	Myc	1	0	0	0	1	1	0	0	0
		MBS	2	1	1	1	0	1	0	2	0
Others	Others	CAG-motif	1	0	0	0	0	0	0	0	0
		ABRE4	1	2	0	0	4	0	0	1	4
		TATA	1	0	1	1	0	0	1	0	0
		ABRE3a	1	2	0	0	4	0	0	1	4
		AT~TATA-box	19	17	0	8	8	15	2	5	17
		CAAT-box	39	55	32	49	35	42	38	35	45
		AAGAA-motif	1	1	0	1	1	3	2	2	2
		as-1	3	3	5	0	1	3	1	1	3
		AT-rich element	1	0	0	0	0	0	0	1	0
		TATA-box	79	73	26	75	52	66	29	66	87
		MYB-like sequence	1	3	3	2	1	0	1	0	2
		CTAG-motif	0	0	1	0	0	1	0	0	0
		box S	0	0	0	1	0	0	2	0	0
		HD-Zip 3	0	0	0	0	1	0	0	0	0
		GC-motif	0	0	0	0	1	0	0	0	0
		CCGTCC motif	0	0	0	0	0	1	0	0	0
		CCGTCC-box	0	0	0	0	0	1	0	0	0
		A-box	0	0	0	0	0	1	0	0	0
		3-AF3 binding site	0	0	0	0	0	1	0	0	0
		AP-1	0	0	0	0	0	0	1	0	0
		ABRE2	0	0	0	0	0	0	0	1	1
		ATC-motif	0	0	0	0	0	0	0	0	1
		AT~ABRE	0	0	0	0	0	0	0	0	4
