# Supplementary material for: Characterization of Carotenoid Cleavage Oxygenase Genes in Cerasus humilis and Functional Analysis of ChCCD1
Source: Plants (Basel). 2023 May 26;12(11):2114. doi: 10.3390/plants12112114 (PMC10255781; doi:10.3390/plants12112114)
Supplement: Supplementary file 1 [file plants-12-02114-s001.zip › Figure S5.pdf]

	ChCCD-like-a	*ChCCD-like-b*	*ChCCD1*	*ChCCD4*	*ChCCD7*	*ChCCD8*	*ChNCED1*	*ChNCED5*	*ChNCED6*
BBR-BPC	34	5	10	7	103	11	75	63	70
MIKC_MADS	9	20	16	18	36	28	28	30	47
Dof	12	45	19	37	32	75	81	32	63
MYB_related	4	12	8	10	18	2	6	2	10
AP2	1	12	6	3	35	10	11	15	25
ZF-HD	0	5	1	7	3	12	6	14	5
GRAS	5	2	5	5	29	6	10	15	20
TCP	4	24	37	113	23	27	29	15	8
bHLH	44	11	15	10	3	13	73	73	168
SRS	0	0	1	2	0	0	0	0	0
C2H2	11	25	44	14	58	24	40	31	26
MYB	45	44	35	34	17	9	36	58	28
B3	2	22	7	2	4	9	11	5	18
bZIP	21	13	39	1	14	63	39	33	114
GATA	6	0	8	5	0	2	10	4	14
ERF	6	24	127	11	103	127	126	37	23
LBD	5	0	7	0	10	6	5	3	1
WOX	0	2	0	0	1	2	4	12	2
CPP	6	1	1	6	7	7	4	4	8
Trihelix	1	5	13	3	0	13	4	5	6
NAC	8	14	26	23	9	15	53	45	8
BES1	0	8	1	0	8	0	16	11	41
WRKY	36	4	26	4	1	9	34	2	25
CAMTA	0	1	5	0	0	0	6	2	3
HD-ZIP	4	8	15	9	4	2	10	0	11
Nin-like	3	2	2	0	2	3	2	2	1
SBP	9	0	2	4	3	6	1	2	3
HSF	3	0	0	1	8	5	0	1	0
ARF	1	2	4	1	1	5	0	2	1
GeBP	4	1	0	0	3	0	0	0	1
G2-like	9	5	1	1	14	15	6	2	2
NF-YB	0	2	1	0	1	0	1	0	0
C3H	0	0	0	2	0	2	5	0	1
EIL	0	0	1	1	0	1	0	0	0
E2F/DP	0	1	7	2	1	2	0	2	1
RAV	1	1	4	0	1	3	0	2	1
VOZ	0	0	0	0	0	0	1	2	0
LFY	2	0	0	0	0	0	0	0	0
S1Fa-like	0	0	0	0	1	1	0	0	0
ARR-B	1	0	3	0	0	0	1	1	0
FAR1	0	0	0	2	0	0	2	0	3
GRF	2	0	0	0	0	1	0	0	0
YABBY	1	0	1	1	0	0	0	1	0
