# Supplementary figures and images for: Characterization of Carotenoid Cleavage Oxygenase Genes in Cerasus humilis and Functional Analysis of ChCCD1
Source: Plants (Basel). 2023 May 26;12(11):2114. doi: 10.3390/plants12112114 (PMC10255781; doi:10.3390/plants12112114)

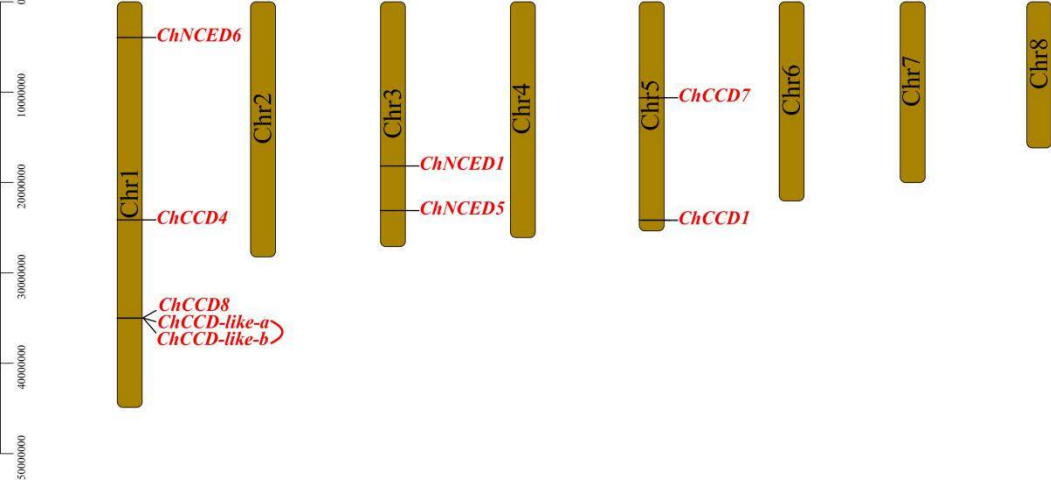

Supplement: Supplementary file 1 [file plants-12-02114-s001.zip › Figure S2.pdf]

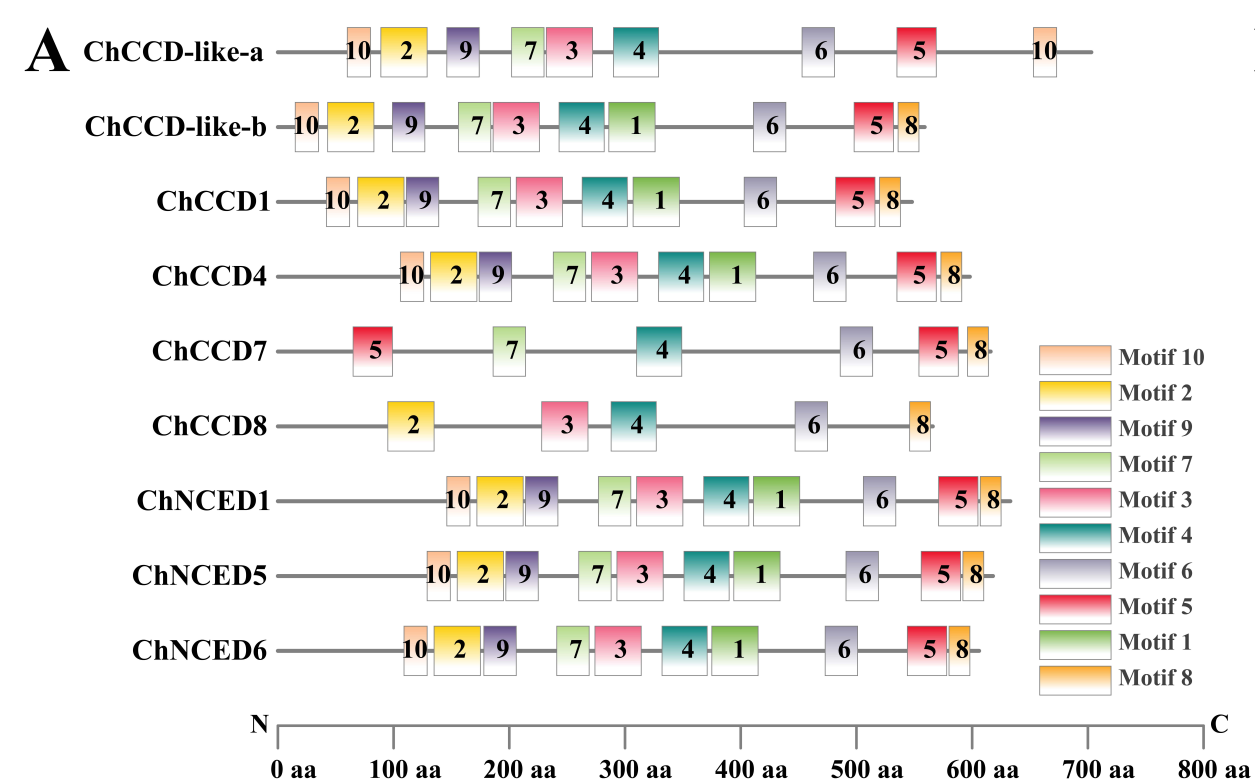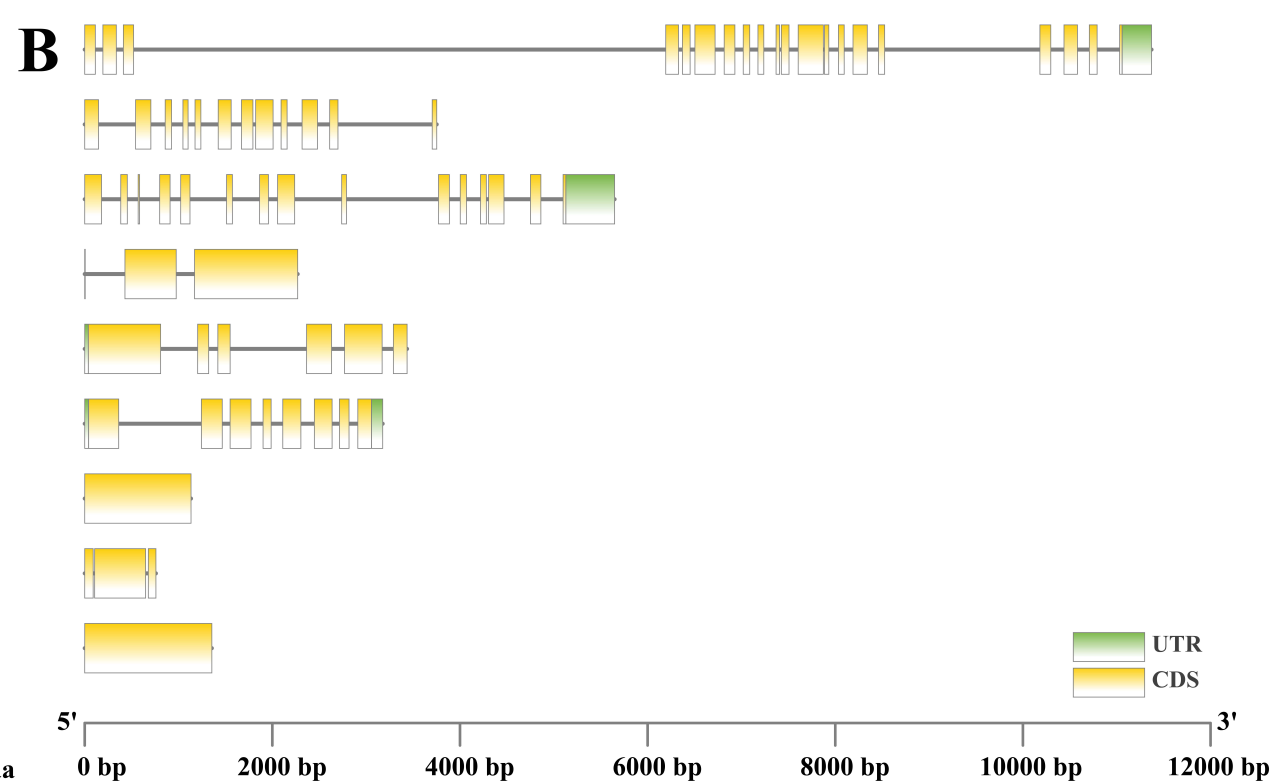

Supplement: Supplementary file 1 [file plants-12-02114-s001.zip › Figure S3.pdf]

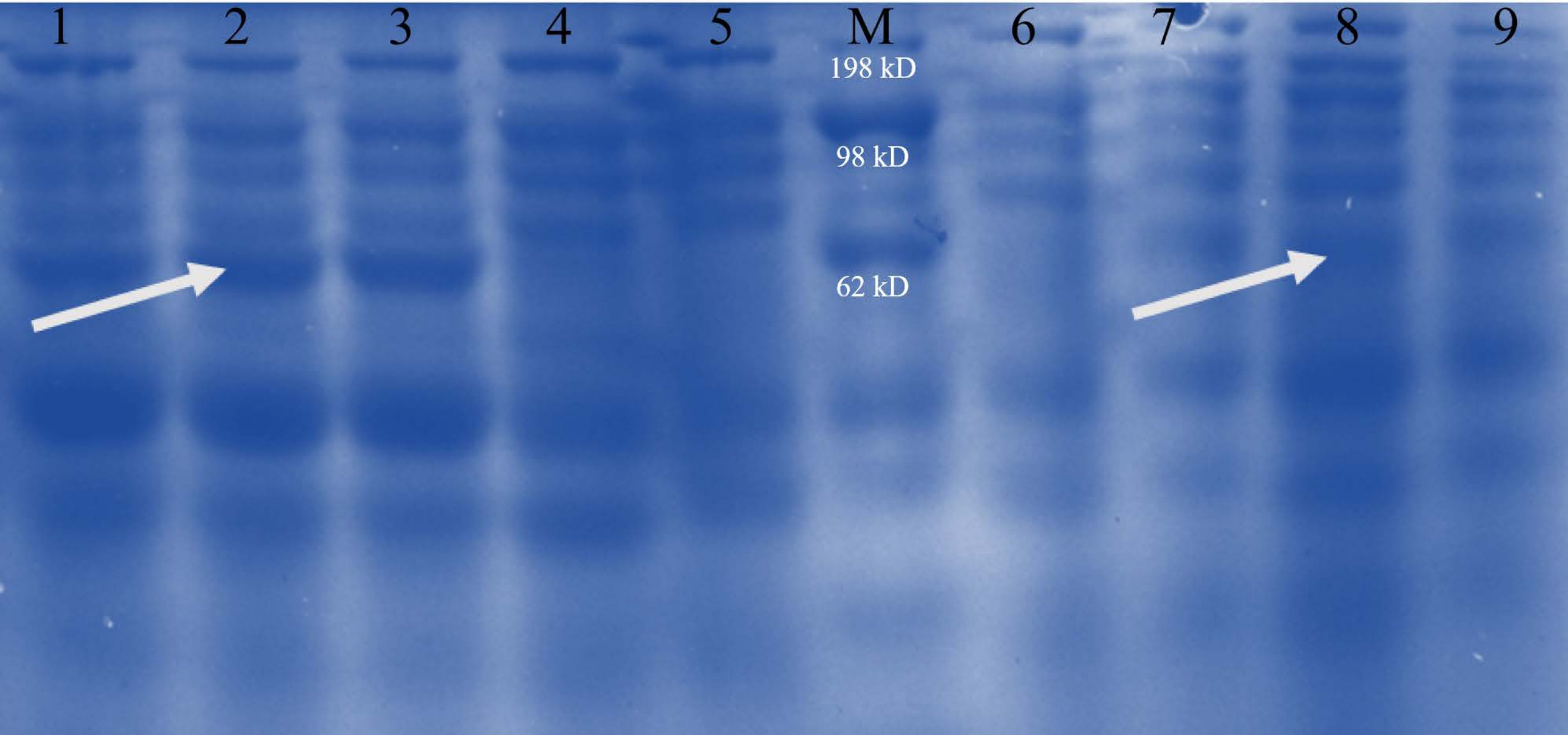

Supplement: Supplementary file 1 [file plants-12-02114-s001.zip › Figure S6.pdf]
